# Mechanical stimulation of induced pluripotent stem cell derived cardiac fibroblasts

**DOI:** 10.1038/s41598-024-60102-w

**Published:** 2024-04-29

**Authors:** Fjodor T. Bekedam, Rowan Smal, Marisa C. Smit, Jurjan Aman, Anton Vonk-Noordegraaf, Harm Jan Bogaard, Marie José Goumans, Frances S. De Man, Aida Llucià-Valldeperas

**Affiliations:** 1 PHEniX Laboratory, Department of Pulmonary Medicine, Amsterdam UMC Location Vrije Universiteit Amsterdam, De Boelelaan 1117, Amsterdam, The Netherlands; 2 Amsterdam Cardiovascular Sciences, Pulmonary Hypertension and Thrombosis, Amsterdam, The Netherlands; 3 Department of Cell and Chemical Biology, Leiden UMC, 2300 RC Leiden, The Netherlands

**Keywords:** Mechanotransduction, Cardiovascular biology

## Abstract

Cardiac fibrosis contributes to the development of heart failure, and is the response of cardiac fibroblasts (CFs) to pressure or volume overload. Limiting factors in CFs research are the poor availability of human cells and the tendency of CFs to transdifferentiate into myofibroblasts when cultured in vitro. The possibility to generate CFs from induced pluripotent stem cells (iPSC), providing a nearly unlimited cell source, opens new possibilities. However, the behaviour of iPSC-CFs under mechanical stimulation has not been studied yet. Our study aimed to assess the behaviour of iPSC-CFs under mechanical stretch and pro-fibrotic conditions. First, we confirm that iPSC-CFs are comparable to primary CFs at gene, protein and functional level. Furthermore, iPSC-derived CFs adopt a pro-fibrotic response to transforming growth factor beta (TGF-β). In addition, mechanical stretch inhibits TGF-β-induced fibroblast activation in iPSC-CFs. Thus, the responsiveness to cytokines and mechanical stimulation of iPSC-CFs demonstrates they possess key characteristics of primary CFs and may be useful for disease modelling.

## Introduction

Heart failure is the most common cause of cardiovascular death, with a 5-year mortality above 50%^1^. One of the pathological changes resulting in heart failure is cardiac fibrosis^2^. Cardiac fibrosis is one of the primary responses to acute injury (replacement fibrosis), such as cardiac infarction, and to chronic stress (interstitial fibrosis), such as pressure overload. Pressure overload-induced cardiac fibrosis is commonly seen in systemic hypertension and pulmonary hypertension, in the left and right ventricles, respectively^3,4^. The resulting interstitial cardiac fibrosis is characterized by accumulation and qualitative changes of the extracellular matrix (ECM). As a result, the passive stiffness of the ventricle increases and its relaxation is impaired^5–7^. Even though cardiac fibrosis plays a significant role in heart failure, current therapies are unable to reverse cardiac fibrosis^8^.

Cardiac fibrosis involves the activation of cardiac fibroblasts (CFs)^9^. Under physiological conditions, CFs are responsible for the homeostasis of the ECM in the heart. The cardiac ECM is dynamic and involves a constant balance between the production and degradation of ECM proteins. ECM proteins are produced as monomers and crosslinked to form strong fibers. Degradation of ECM proteins is mediated by a balance between matrix metalloproteinases and their inhibitors. Under pathological conditions, such as pressure overload, CFs are further activated and will transdifferentiate into cardiac myofibroblasts. Myofibroblasts are characterized by increased production and deposition of ECM proteins along with an upregulated expression of α-smooth muscle actin (α-SMA). The transdifferentiation of CFs into myofibroblasts is heavily regulated by the transforming growth factor beta (TGF-β) pathway and mechanotransduction^3^. In fact, stiffness of the cellular environment, shear stress and mechanical strain can be sensed through mechanosensitive complexes and affect the transdifferentiation^10^. While the importance of mechanical stimuli has been acknowledged, studying the behaviour of CFs in vitro and developing anti-fibrotic treatments has remained challenging.

The in vitro study of cardiac fibrosis is limited by the poor availability of primary CFs and the fact that primary CFs quickly transdifferentiate into myofibroblasts when cultured on stiff, plastic culture plates and in the presence of fetal bovine serum^11,12^. This limits the time cells isolated from tissue can be used to address scientific questions. In addition, when studying the development of heritable heart diseases, healthy tissue to isolate fibroblasts from is often not available. When CFs are isolated from diseased tissue, human primary CFs are commonly in a pre-activated state and may not represent the earlier stages of disease when fibrosis has not yet reached an irreversible end-stage. Therefore, instead of using primary CFs, induced pluripotent stem cells (iPSC)-derived CFs may offer a suitable alternative. iPSCs are commonly used to generate various cell types, such as cardiomyocytes, to model diseases. Recently, several protocols have been published to generate CFs^13–15^, providing the scientific community with a powerful tool to study the development of cardiac fibrosis. Although iPSC-CFs have been characterized and compared to primary CFs, their behaviour under mechanically dynamic conditions has not been investigated yet. It has been shown that cyclic stretch of primary CFs at physiological levels can alter the response to biochemical stimuli^16–18^. However, it is not known whether iPSC-CFs possess a similar kind of mechanosensitive response. To answer this question, this study aimed to study the effect of mechanical and pro-fibrotic stimulation on iPSC-derived CFs.

## Results

### Comparable gene expression of cardiac fibroblast markers and gel contraction in iPSC-CFs and primary CFs

**Figure 1:**
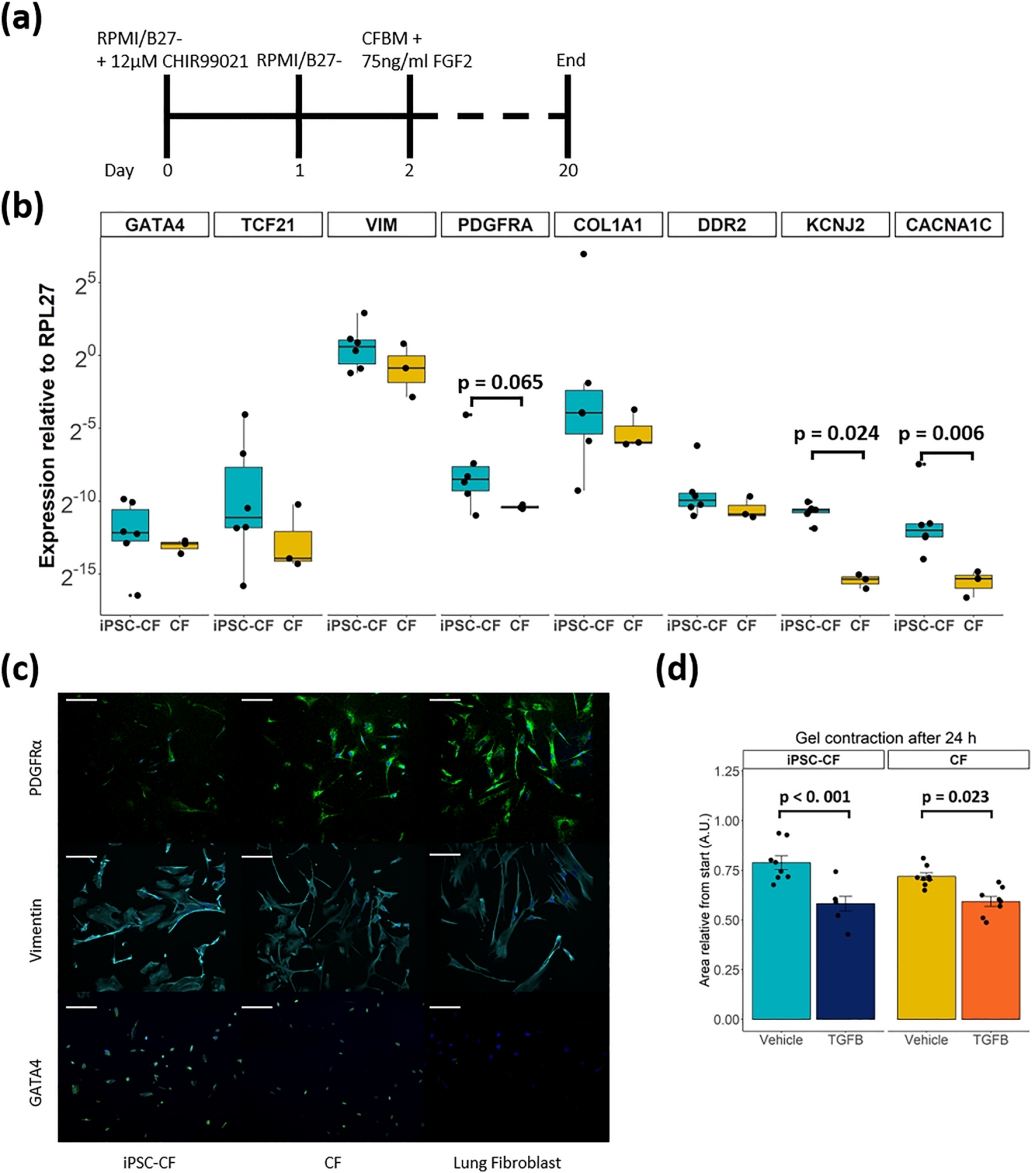
(**a**) Overview differentiation protocol. CFBM indicates cardiac fibroblast basal medium and FGF2 fibroblast growth factor 2. (**b**) Gene panel of cardiac (*GATA4, TCF21*), fibroblast (*VIM, PDGFRA, COL1A1, DDR2*) and ion channel (*KCNJ2, CACNA1C)* genes (N = 6 differentiations, N = 3 replicates from 1 donor). A student’s t test is performed to test difference in gene expression between the two groups. In the case of *KCNJ2* a Mann–Whitney U test was applied. (**c**) Immunostaining for PDGFRα, Vimentin and GATA4 in iPSC-CF, CF and lung fibroblast. Counterstained with Hoechst in blue. Scale bar indicates 100 μm. (**d**) Gel contraction assay, gels stimulated with vehicle or TGF-β for 24 h. Data is presented as area relative from 0 h (N = 8 gels per group). A linear regression model was performed to test for difference between stimulation and cell type, with a Tukey’s post hoc analysis. See also Fig. S1. Graphs represent mean ± SEM.

We generated iPSC-CFs following the protocol established by Zhang et al.^13^ (Fig. 1a). To ensure differentiation of iPSCs into iPSC-CFs the cells were characterized at the gene, protein and functional level. At the end of differentiation, the cells expressed markers associated with the cardiac lineage, such as the genes encoding GATA4 (*GATA4*) and transcription factor 21 (*TCF21*). In addition, fibroblast genes *VIM*, *PDGFRA*, *COL1A1* and *DDR2* were expressed at comparable levels as primary CFs (Fig. 1b). Gene expression of ion channels involved in the conductance of the cardiac action potential (*KCNJ2, CACNA1C)* was higher in iPSC-CFs compared to primary CFs. The morphology of the iPSC-CFs was spindle-shaped and comparable to primary CFs. At the protein level, iPSC-CFs expressed the common fibroblast markers PDGFRα and vimentin, as do primary CFs and primary lung fibroblasts. Confirming their cardiac identity, iPSC-CFs as well as primary CFs showed nuclear expression of the cardiac transcription factor GATA4 while this transcription factor was absent on primary lung fibroblasts (Fig. 1c). To characterize the functionality of iPSC-CFs a gel contraction assay was performed, which is indicative for cell-ECM interaction. Similar to primary CFs, the iPSC-CFs contracted the gel which was further increased when stimulating with TGF-β (Fig. 1d, Fig. S1). Overall, our data confirms that we generated functional CFs from human iPSCs which are comparable to primary human CFs.

### Stretch and TGF-β co-stimulation reduced collagen 1 expression in iPSC-CFs

To study how iPSC-CFs behave in a mechanically dynamic environment like the heart, cells were exposed to 10% cyclic stretch at 1 Hz for 72 h (Fig. S2). This resembles the strain that cells experience under physiological conditions in a human heart at rest^16,18,19^. In addition, cells were treated with TGF-β as a pro-fibrotic stimulus. Collagen 1 expression, the most abundant component of the cardiac ECM^20^, was investigated.

**Figure 2:**
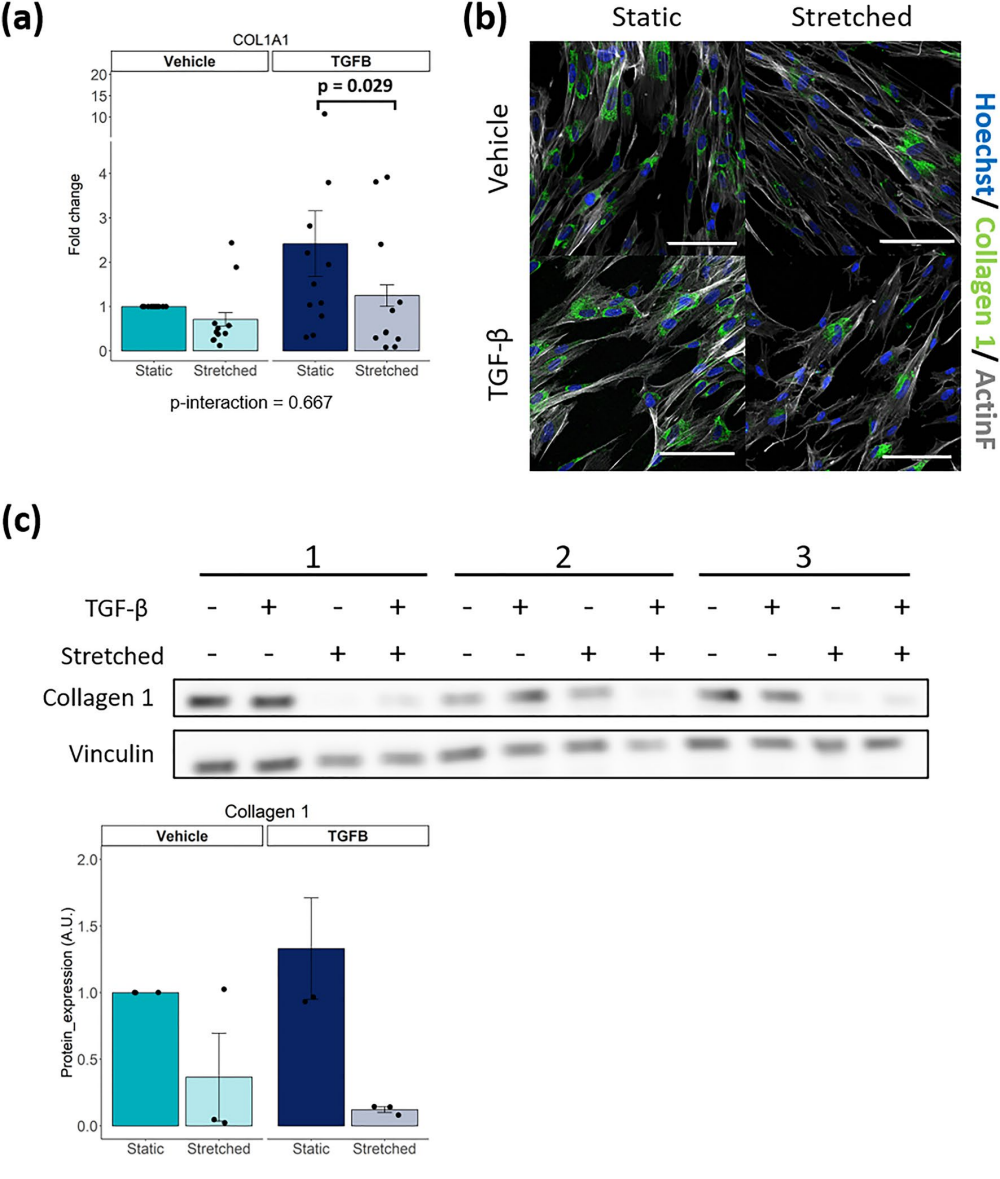
(**a**) Gene expression of *COL1A1* after stimulation of iPSC-CF with TGF-β and cyclic stretch (N = 11 from 6 independent differentiation batches). (**b**) Immunofluorescent staining of iPSC-CF for collagen 1, counterstained with Hoechst and F-actin. (**c**) Collagen 1 protein measured and quantified using western blot in iPSC-CF after stimulation with TGF-β and cyclic stretch (N = 3). A linear mixed model, with the experimental conditions considered as fixed parameters and the batches of differentiation as random parameters was applied. Tukey post-hoc tests were performed to correct for multiple comparisons. Collagen 1 protein expression was statistically tested using the non-parametric Kruskal–Wallis test. Graphs represent mean ± SEM.

Exposing iPSC-CFs to cyclic stretch did not alter *COL1A1* gene expression (Fig. 2a). Stimulation with TGF-β showed a clear trend of *COL1A1* upregulation, while cyclic stretch in the presence of TGF-β significantly reduced the expression of *COL1A1*. Immunofluorescence and western blot showed that stretch reduced collagen 1 protein expression in iPSC-CF, both in the presence and absence of TGF-β (Fig. 2b, c, Fig. S3). To summarize, cyclic stretch inhibits the expression of collagen 1, especially in the presence of TGF-β.

### Stretch reduces the expression of ECM remodelling genes in iPSC-CFs

One important function of fibroblasts is to maintain ECM homeostasis by regulating the balance between ECM production, degradation and modification through expression of matrix metallopeptidases, their inhibitors and lysyl oxidases, respectively^21^. Therefore, we investigated gene expression of key-players in ECM regulation in iPSC-CF under pro-fibrotic and cyclically stretched conditions.

**Figure 3:**
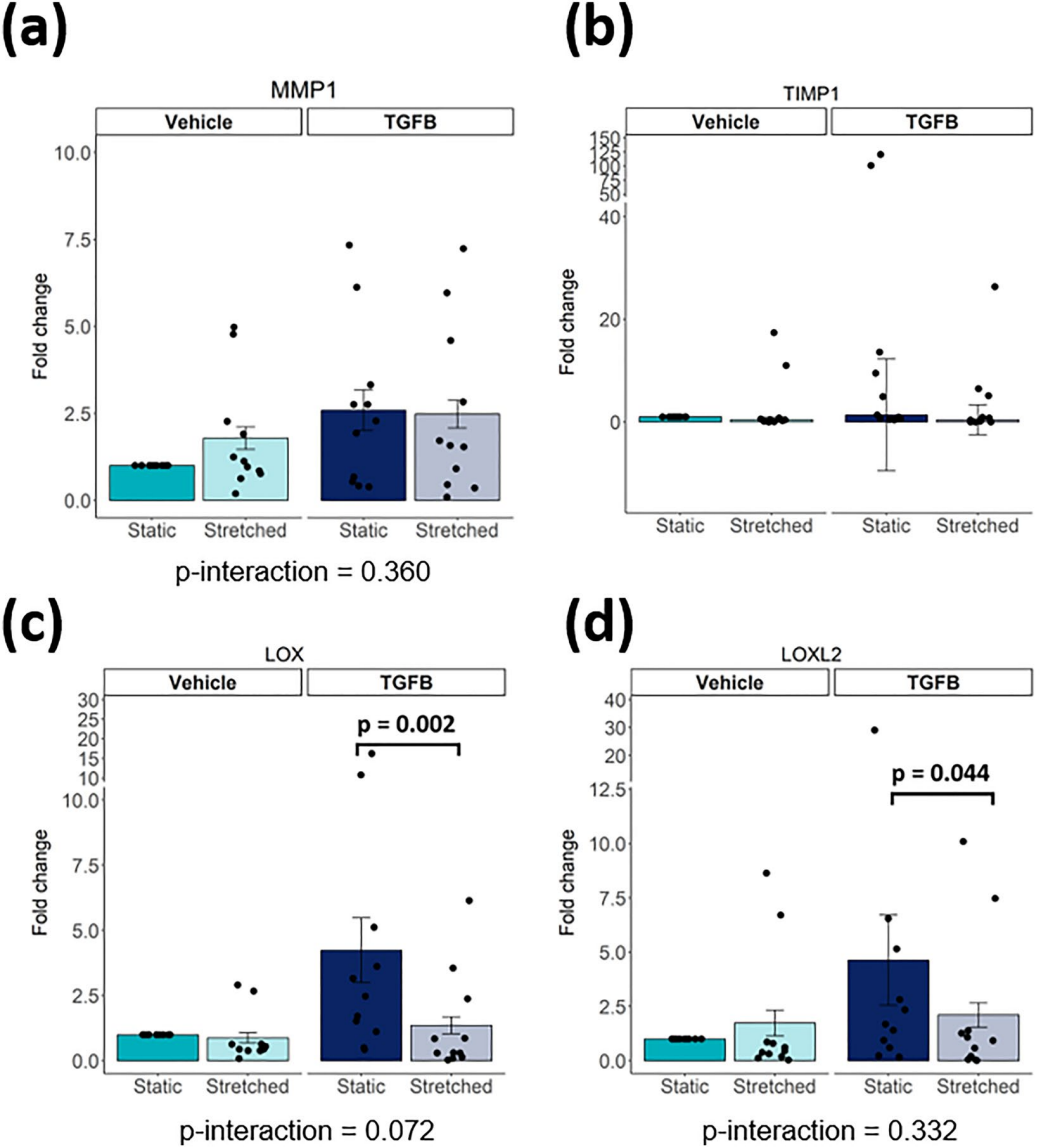
Gene expression of *MMP1, TIMP1, LOX, LOXL2* after stimulation of iPSC-CF with TGF-β and cyclic stretch (N = 11 from 6 independent differentiation batches. For *MMP1, LOX* and *LOXL2* A linear mixed model, with the experimental conditions considered as fixed parameters and the batches of differentiation as random parameters was applied. Tukey post-hoc tests were performed to correct for multiple comparisons. Graphs represent mean ± SEM. For *TIMP1* a Kruskal–Wallis test was performed. Graph represent median ± IQR.

In iPSC-CFs, cyclic stretch or TGF-β stimulation did not affect mRNA levels of the matrix metallopeptidase 1 gene (*MMP1*) and neither of its inhibitor, encoded by *TIMP1* (Fig. 3a, b). In unstimulated conditions, *LOX* and *LOXL2*, genes associated with ECM crosslinking, were not affected by stretch, while during TGF-β stimulation cyclic stretched reduced *LOX* and *LOXL2* expression in iPSC-CFs (Fig. 3c, d).

### Cyclic stretch inhibits TGF-β induced transdifferentiation of iPSC-CF into myofibroblasts

Pathologic conditions such as pressure overload and the presence of pro-fibrotic cytokines will activate CFs and transdifferentiate them into myofibroblasts^3^. This transdifferentiation is most commonly marked by an upregulation of α-smooth muscle actin (α-SMA). To investigate cardiac fibroblast transdifferentiation, iPSC-CFs were exposed to cyclic stretch (Fig. S2) and/or stimulated with TGF-β.

**Figure 4:**
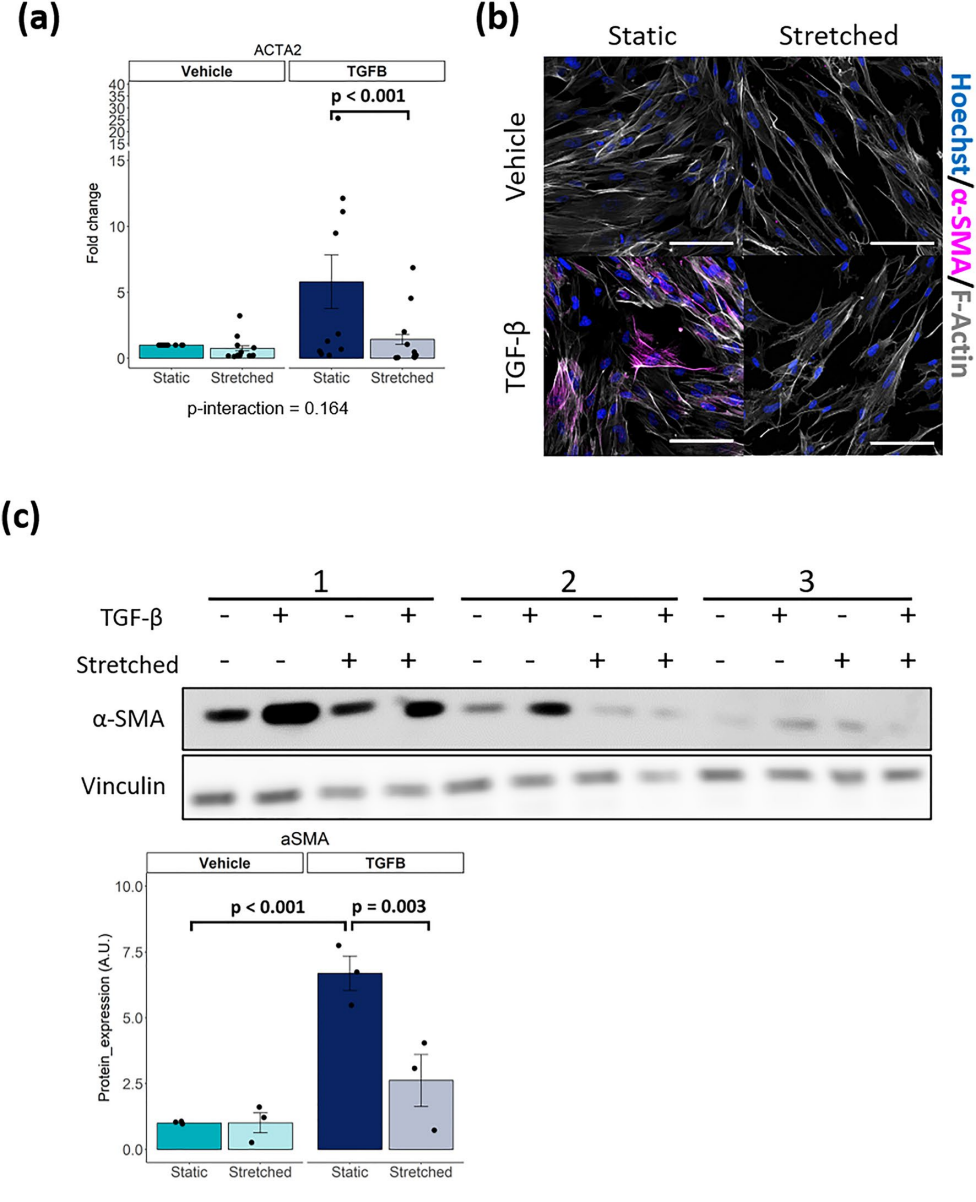
(**a**) Gene expression of *ACTA2* after stimulation of iPSC-CF with TGF-β and cyclic stretch (N = 11 from 6 independent differentiation batches. (**b**) Immunofluorescent staining of iPSC-CF for α-SMA, counterstained with hoechst and F-actin. Scale bar indicates 100 μm. (**c**) α-SMA protein measured and quantified using western blot in iPSC-CF after stimulation with TGF-β and cyclic stretch (N = 3). A linear mixed model, with the experimental conditions considered as fixed parameters and the batches of differentiation as random parameters was applied. Tukey post-hoc tests were performed to correct for multiple comparisons. Graphs represent mean ± SEM.

Cyclic stretch had no effect on *ACTA2* expression, the gene encoding α-SMA (Fig. 4a). TGF-β stimulation promoted induction of *ACTA2 *expression, although high variation per iPSC-CF batch was observed. Co-stimulation of cyclic stretch and TGF-β resulted in a significant reduction of *ACTA2* expression compared to the static condition with TGF- β stimulation. iPSC-CFs present with very low basal levels of α-SMA protein and no α-SMA stress fibres were observed under static and stretched conditions (Fig. 4b, c, Fig. S3). In line with the mRNA data, cyclic stretch alone had no effect on α-SMA protein expression. When stimulated with TGF-β, α-SMA protein levels increased, and immunofluorescence imaging revealed stress fibre formation under static conditions. Stimulation with TGF-β in combination with cyclic stretch significantly reduced α-SMA protein expression and stress fibres formation in iPSC-CFs. To conclude, TGF-β induced transdifferentiation of iPSC-CFs into myofibroblasts can be inhibited by cyclic stretch.

### Cyclic stretch inhibits TGF-β induced signalling in iPSC-CFs

**Figure 5:**
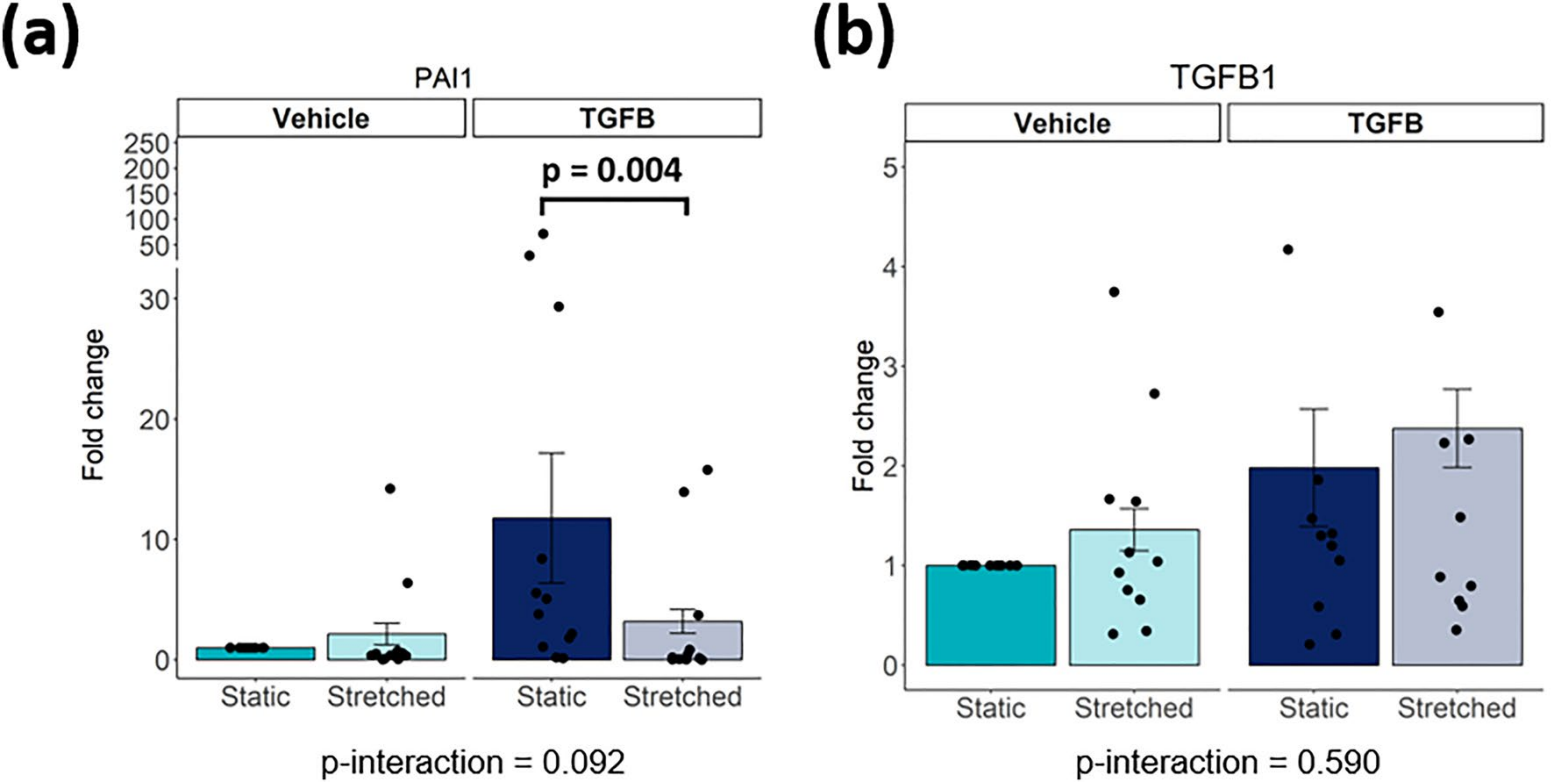
(**a**, **b**) Gene expression of *PAI1, TGFB1* after stimulation of iPSC-CF with TGF-β and cyclic stretch (N = 11 from 6 independent differentiation batches). A linear mixed model, with the experimental conditions considered as fixed parameters and the batches of differentiation as random parameters was applied. Tukey post-hoc tests were performed to correct for multiple comparisons.

TGF-β signalling is one of the most well-studied pathways involved in fibroblast activation. In order to investigate whether this pathway is activated in response to mechanical stimulation, *PAI1* and *TGFB1* gene expression were analysed. The *PAI1* gene is a direct target of the transcription factors downstream TGF-β signalling, and commonly used as a marker for TGF-β pathway activation^22^. Under static conditions, TGF-β stimulation increased *PAI1* gene expression (not significant), while cyclic stretch abrogated TGF-β-induced *PAI1* expression (Fig. 5a). TGF-β stimulation or cyclic stretch did not alter *TGFB1* expression (Fig. 5b), indicating that the effect of mechanical stimulation on the TGF-β pathway is not regulated at the gene level, but may be regulated at the protein level instead. In conclusion, cyclic stretch can inhibit TGF-β induced gene expression independent of *TGFB1* expression.

## Discussion

CFs are the main contributors of cardiac fibrosis development^3^. The availability of human CFs is limited hampering the field to move forward. To date, CFs can be generated from iPSCs, which could provide an unlimited source of human CFs^13–15^. However, the behaviour of iPSC-CFs in relation to mechanical stimulation had not been investigated yet.

In this study we demonstrated that iPSC-CFs are comparable to primary CFs with regard to the expression of key CF markers at gene and protein levels. Expression of the cardiac markers *GATA4* and *TCF21* indicate the cardiac lineage of the cells. Furthermore, expression of the mesenchymal markers *VIM and PDGFRA* as well as the ECM component *COL1A1* and the collagen binding receptor *DDR2* support their fibroblast phenotype. In addition, we showed that iPSC-CFs respond to pro-fibrotic and mechanical stimulation. TGF-β induces CFs transdifferentiation into myofibroblasts and promotes ECM remodelling. Mechanical stimulation in the form of cyclic stretch at physiological levels reduces collagen expression in iPSC-CFs. Interestingly, cyclic stretch also protects against TGF-β stimulation, preventing the cells from transdifferentiating into myofibroblasts.

One can only use iPSC-derived cells when they accurately represent their primary counterparts. Key characteristics of CFs are a defined mRNA profile, responsiveness to pro-fibrotic cytokines, interaction with the ECM and mechanical sensitivity. iPSC-CFs generated using the protocol developed by Zhang et al. showed a comparable RNA sequencing profile in iPSC-CFs and primary CFs^13^. Using our iPSCs lines, following the same protocol we generated iPSC-CFs with an mRNA profile comparable to primary CFs. Furthermore, at a functional level we demonstrated that iPSC-CFs interact with their environment in a similar way as primary CFs, and respond to pro-fibrotic stimulation. These results indicate that iPSC-CFs possess several key characteristics of primary CFs and may be suitable to investigate the behaviour of CFs and develop disease models of cardiac fibrosis.

In order to investigate the behaviour of CFs in their native environment, we next investigated the behaviour of iPSC-CFs under physiologically relevant conditions. In an effort to mimic the dynamic environment of the continuously beating heart, we investigated the effects of cyclic mechanical stretch on iPSC-CFs. The importance of mechanical stimulation has been acknowledged, but the effects of mechanical stimulation on CFs remain controversial in in vitro studies^23^. On one hand, it has been reported that cyclic stretch may induce transdifferentiation of CFs into myofibroblasts. On the other hand, it has been shown that cyclic stretch may have a protective effect instead. One of the main factors influencing this controversy is the usage of cell sources from different species.

As primary human CFs are limited in availability, iPSC-CFs could provide a representative and stable source of cells to move forward. In order to study how iPSC-CFs and primary CFs behave in a mechanically dynamic environment similar to the heart, cells were exposed to 10% cyclic stretch at 1 Hz for 72 h^19^. With this approach, we demonstrated that: Cyclic stretch alone inhibits expression of collagen 1 but does not affect iPSC-CFs transdifferentiation or expression of matrix remodelling genes. In addition, cyclic stretch is protective against TGF-β mediated myofibroblast transdifferentiation in iPSC-CFs, resulting in normalised expression of collagen 1, α-SMA and matrix remodelling genes such as *TIMP1* and *MMP1*.

The cause of the aforementioned controversy in literature regarding either the pro-fibrotic or anti-fibrotic response of CFs to mechanical stimulation is hard to pin-point; experimental conditions vary widely between studies, such as cell origin, the duration of the experiment, the surface coating and the presence of serum. A common trend in all those studies is that there may be a time-dependent response of stretch. It was shown in primary mouse CFs that the response starts with an initial increase in phosphorylation of AKT, a downstream kinase involved in the transduction of mechanical stimuli^24,25^. At the gene level, it was shown in primary rat CFs that there is an initial increase in fibrotic markers (i.e. *ACTA2, TGFB1, CTGF)* after 4 h followed by a reduced increase after 24 h^26^. Roche et al. observed a similar effect in primary rat CFs with an apparent reduced increase of *COL1A1* gene expression after 48 h compared to 24 h^27^. 72 h of cyclic stretch was instead shown to inhibit TGF-β induced fibroblast activation in primary human CFs^16,18^. Furthermore, it has been demonstrated that 96 h of cyclic stretch can promote or inhibit the response of primary mouse CFs to a broad spectrum of biochemical stimuli, including TGF-β, angiotensin II, interleukin-1β and others^17^. Overall, it appears that longer stimulation results in a gradual decrease of an initial pro-fibrotic response with eventually cells balancing the fibrotic response to the mechanically active environment in order to reach homeostasis. We may hypothesize that the duration of this response curve is dependent on different factors, including the origin and age of the cells, their culture conditions (surface coating, substrate stiffness, or medium supplementation with serum) and the presence of other cell types^23^. A clear association between mechanosensing and a response of CFs is apparent, but there is a need for a reproducible cell type to better understand this phenomenon.

TGF-β signalling is one of the main pathways involved in the activation of CFs and development of cardiac fibrosis^28^. Exposure of iPSC-CFs to TGF-β promotes the expression of fibrotic and myofibroblast markers, such as α-SMA. When stretched however, this effect is diminished. How mechanical changes communicate with the TGF-β pathway is not well understood. On one hand, mechanical strain has been shown in tissue to release active TGF-β from the ECM, which would promote fibroblast activation^29^. On the other hand, in this in vitro study mechanical strain appears to inhibit fibroblast activation, indicating that there may be other mechanisms at play in this model. It is unknown whether this anti-fibrotic effect is directly caused by interplay between mechanosensitive complexes and the TGF-β pathway. Mechanosensitive receptors such as integrins or mechanoresponsive factors such as YAP/TAZ may communicate with the TGF-β pathway^30,31^. Alternatively, cyclic stretch may have an indirect effect, for example through internalization of extracellular receptors, altering the response to ligand stimulation. Regardless, the field of mechanotransduction in CFs remains requires further investigation.

While iPSCs have started a new era of research, the usage of these cells comes with limitations. iPSC-CFs showed many similarities with primary CFs, but the maturity of iPSC-derived cell lineages remains an important topic of contention. Although maturation is clearly defined for some cell types, such as cardiac myocytes, a clear definition lacks for CFs. The heterogeneity and plasticity of this cellular population under physiological conditions makes it difficult to set well defined standards of “mature” CFs^32^. iPSC-CFs present with various characteristics of primary cells, but they differ in several aspects as well. For example, Zhang et al. noted an increased proliferation capacity in iPSC-CFs and foetal CFs compared to adult CFs, indicating the iPSC-CFs may be more foetal-like^13^. This increased proliferation capacity and ability to stay in an inactivated state while in culture increases the applicability of the iPSC-CFs in research, as it has been demonstrated that CFs which have transdifferentiated into myofibroblast will have an altered response to mechanical stimulation^33^. In addition, little is known about the electrophysiological characteristics of iPSC-CFs and their interaction with other conducting cells such as cardiomyocytes^34^. Further electrophysiological characterisation should be performed to better understand the behaviour of these cell in the electrical circuit of the heart.

To conclude, in this study we demonstrated that iPSC-derived CFs show similar gene and protein expression as primary CFs. In addition, pro-fibrotic stimulation promoted transdifferentiation of iPSC-CFs into a myofibroblast phenotype. When stimulated with cyclic stretch, this transdifferentiation is inhibited. Together, the mechano- and TGF-β-responsive characteristics support the use of iPSC-CFs for physiological relevant disease modelling. Future studies could further dive into the mechanisms driving cardiac fibroblast behaviour and cardiac fibrosis.

## Methods

### Cell culture

Three human iPSCs lines were derived from one healthy female subject generated as described before^35^. iPSCs were cultured on vitronectin XF (StemCell Technologies) coated plates in TeSR-E8 medium (StemCell Technologies). Cells were passaged every 7 days in a 1:10 ratio using 0.5 µM EDTA solution (Invitrogen) at RT for 5 min and manual dissociation. Commercially available human primary ventricular cardiac fibroblasts (Lonza) from 1 healthy donor were cultured according to the manufacturer’s instructions. Primary cardiac fibroblasts were used for a maximum of 3 passages. Mycoplasma tests were routinely performed for all cell cultures in this study.

### Cardiac fibroblast differentiation

For the generation of iPSC-CFs a protocol developed by Zhang et al. was used^13^. Briefly, human iPSCs were dissociated with 1 mL/well 0.5 µM EDTA solution (Invitrogen) at RT for 5 min and seeded on Vitronectin XF (StemCell Technologies) coated 6-well plates at a density of 15.000–30.000 cells/cm^2^ in TeSR-E8 medium (StemCell Technologies) supplemented with 5 μM ROCK inhibitor (Y-27632) (Tocris) for 24 h. Cells were cultured for 6–7 days in TeSR-E8 medium with medium changes every other day until they reached 100% confluency and differentiation started (day 0). At day 0, the medium was changed to 2.5 mL/well RPMI + B27 without insulin (Gibco) and supplemented with 12 µM CHIR99021 (Tocris) for 24 h (day 1). After day 1, the medium was changed to 2.5 mL RPMI + B27 without insulin for 24 h (day 2). Afterwards, the medium was changed to 2.5 mL/well of the CFBM medium (Table S1) supplemented with 75 ng/mL bFGF (StemCell Technologies). Cells were refreshed with 2 mL/well CFBM supplemented with 75 ng/mL bFGF every other day until day 20 when RNA was collected, and cells were dissociated using TrypLE Select (10x) (Thermo Fisher) for 10 min at 37 °C. After dissociation, cells were cultured in DMEM + 10% Fetal bovine serum. For the first two passages, 5 μM ROCK inhibitor was added for 24 h to help cell attachment. Cells between passage 3–6 were used for experiments.

### Immunofluorescent staining

Cells were fixed in 4% paraformaldehyde in phosphate-buffered saline (PBS) for 20 min at room temperature. Samples were permeabilized, blocked, and incubated at 4 °C overnight with primary antibodies against GATA4 (1:200, Abcam, ab124265), PDGFR-α (1:250, Cell Signaling Technology, #3174), vimentin (1:500, Abcam, ab73159) or α-SMA–Cy3-conjugated (1:400, Sigma, C6198). Samples were then incubated for 1 h at room temperature with secondary antibodies conjugated with Alexa-488, Alexa-555, or Alexa-647 (1:500; Abcam). The cells were counterstained with Hoechst 33342 nuclear dye (1:500; Santa Cruz Biotechnology) and actiStain phalloidin conjugated to Alexa-670 (1:200; Cytoskeleton Inc.).

Images were captured under a laser confocal microscope (Nikon A1R, Nikon; RCM1, confocal.nl) and analysed on ImageJ (NIH).

### Quantitative real-time PCR analysis

Total RNA was extracted using the RNeasy® Mini Kit with DNAse I digestion (Qiagen) following the manufacturer’s protocol. The concentration and purity of the RNA were measured on the Nanodrop One spectrophotometer (Thermofisher Scientific). Reverse transcription to cDNA was carried out using the iScript™ cDNA Synthesis Kit (Bio-Rad). Quantitative real-time PCR amplifications were performed with 10 ng (2 µL) of cDNA in a final volume of 10 µL, containing 5 µL of Fast SYBR Green MasterMix (Thermofisher Scientific), 2 µL of RNase-free PCR-grade water (Thermofisher Scientific), and 1 µL of both forward and reverse 10 μM primer solutions (sequences specified in Table S2). Housekeeping gene ribosomal protein L27 (RPL27) was used to ensure the validity and reproducibility of the results. Data were collected and analyzed in duplicate on the CFX384™ Real-Time System (C1000 Touch™ Thermal Cycler, Bio-Rad). The Livak method was used to quantify the relative (2^−ΔΔCT^) expression of each gene between groups.

### Western blot

Whole cell lysate samples were collected in lysis buffer (20 mM Tris–HCl, 150 mM NaCl, 100 mM KCl, 2 mM EDTA-NaOH, 5% Igepal, and 0.5% Triton X-100; pH 8.0) supplemented with PhosSTOP™ (Roche) and cOmplete™ Protease Inhibitor Cocktail (Roche) according to manufacturer’s instructions. Lysates were prepared with 1 × NuPage LDS sample buffer (Thermofisher Scientific) and 50 µM DTT (Thermofisher Scientific). Protein samples were loaded on 4–12% NuPage Bis–Tris protein gel (Thermofisher Scientific) and electrophoresed at 200 V for ~ 1 h. Separated proteins were transferred to 0.45 µM Amersham Hybond ECL nitrocellulose membranes (Thermofisher Scientific) and blocked with 5% BSA (Sigma) in Tris-buffered saline (pH 7.6) with 0.1% Tween (TBS-T) for 1 h at room temperature. Membranes were incubated overnight at 4 °C with gentle shaking in either 5% BSA or 5% milk with primary antibodies against collagen 1 (1:2000, Abcam, Ab138492), vinculin (1:1000, Sigma-Aldrich, V9131), α-SMA (1:1000, Dako, M0851). Afterwards, appropriate HRP-conjugated secondary antibodies were incubated in 5% BSA or 5% milk for 1 h at room temperature. Bands were visualized with Amersham ECL Prime Blotting Detection Reagent (GE Healthcare Life Sciences) detected with the Amersham™ Imager 600 (GE Healthcare Life Sciences), quantified with ImageJ (NIH), and normalized to vinculin expressions.

### Gel contraction assay

Cells were mixed with rat tail collagen type 1 (Corning) according to the manufacturer’s instructions to a final concentration of 2 mg/mL, in a final volume of 150 μl and 50.000 cells per gel. The mixture was pipetted onto a glass coverslip in a 6-well plate and allowed to harden for 30 min at 37 °C. Next, 1.5 mL DMEM + 10% fetal bovine serum was added to the well to cover the gel. The following day, the medium was replaced with DMEM and the cells were starved for 6 h. Afterwards, the gels were detached from the coverslips and medium was replaced with either DMEM or DMEM + 10 ng/mL TGF-β1 (Stem Cell Technologies). Pictures were taken after 24 h to monitor the contraction of the gels.

### Mechanical stretch and TGF-β stimulation

Cells were seeded on rat tail collagen I coated BioFlex® Culture Plates (Dunnlab) coated with 5 μg/ml fibronectin (Sigma) according to the manufacturer’s instructions, using DMEM with 10% fetal bovine serum. After 24 h, the medium was changed either to DMEM or DMEM + 10 ng/mL TGF-β1 and cells were subjected to equiaxial cyclic strain in a sinusoidal pattern (0–10%) at 1 Hz for 72 h using a Flexcell FX-6000 Tension straining device (Flexcell, Dunnlab) and compared to static cultures (0%). Strain experiments were performed with 6 different batches of CFs differentiations, using 3 iPSCs clones of one healthy subject.

### Statistical analysis

Statistical analyses were performed with R statistics package version 4.2.3 (R Foundation for Statistical Computing, Vienna, Austria) and R studio version 2023.09.0 Build 463 (R-studio, Boston, MA, USA). Normality of the data was checked using a Shapiro–Wilk test with *p*-values < 0.05 considered as not normal. Data from *KCNJ2* gene expression and collagen 1 protein expression was not normally distributed. Unless otherwise indicates statistics were performed as follows. When comparing two groups, a student’s t-test was applied. In the case of *KCNJ2*, the non-parametric Mann–Whitney U test was applied. When comparing multiple groups, a linear mixed model, with the experimental conditions considered as fixed parameters and the batches of differentiation as random parameters was applied. Tukey post-hoc tests were performed to correct for multiple comparisons. For collagen 1 protein expression the non-parametric Kruskal–Wallis test was performed. *P*-values < 0.05 were considered statistically significant.

### Supplementary Information

Supplementary Information.

## Data Availability

No datasets were generated or analysed during the current study.
