# Author Correction: Mechanical stimulation of induced pluripotent stem cell derived cardiac fibroblasts

**DOI:** 10.1038/s41598-024-64127-z

**Published:** 2024-06-10

**Authors:** Fjodor T. Bekedam, Rowan Smal, Marisa C. Smit, Jurjan Aman, Anton Vonk-Noordegraaf, Harm Jan Bogaard, Marie José Goumans, Frances S. De Man, Aida Llucià-Valldeperas

**Affiliations:** 1 PHEniX Laboratory, Department of Pulmonary Medicine, Amsterdam UMC Location Vrije Universiteit Amsterdam, De Boelelaan 1117, Amsterdam, The Netherlands; 2 Amsterdam Cardiovascular Sciences, Pulmonary Hypertension and Thrombosis, Amsterdam, The Netherlands; 3 Department of Cell and Chemical Biology, Leiden UMC, 2300 RC Leiden, The Netherlands

Correction to: *Scientific Reports* 10.1038/s41598-024-60102-w, published online 29 April 2024

The original version of this Article contained an error in the title of the paper, where the term “cell” was omitted.

The original Article has been corrected.

