# Supplementary material for: Mechanical stimulation of induced pluripotent stem cell derived cardiac fibroblasts
Source: Sci Rep. 2024 Apr 29;14:9795. doi: 10.1038/s41598-024-60102-w (PMC11058244; doi:10.1038/s41598-024-60102-w)
Supplement: Supplementary file 1 — Supplementary Information. [file 41598_2024_60102_MOESM1_ESM.pdf]

Supplemental Information

Table S1: CFBM composition

Component	Catalog nr	Final concentration
DMEM	Thermo Fisher 10566016	Basal medium
GlutaMAX™ Supplement	Thermo Fisher 35050061	1.5 mM
HLL supplement	Lifeline cell technology LS-1001	HSA: 500 µg/mL<br>Linoleic Acid: 0.6 µM<br>Lecithin: 0.6 µg/mL
Ascorbic acid	Sigma, #A-1417	50 µg/mL
Hydrocortisone hemisuccinate	StemCell Technologies # 74142	1.0 µg/mL
Rh Insulin	Sigma, #I9278	5 µg/mL

Table S2: qPCR primer pairs

Primer name	Forward Primer	Reverse Primer
RPL27	ATCGCCAAGAGATCAAAGATAA	TCTGAAGACATCCTTATTGACG
TCF21	TCCTGGCTAACGACAAATACGA	TTTCCCGGCCACCATAAAGG
PDGFRA	TTTTTGTGACGGTCTTGGAAGT	TGTCTGAGTGTGGTTGTAATAGC
COL1A1	GAGGGCCAAGACGAAGACATC	CAGATCACGTCATCGCACAAC
DDR2	GCTATATGCCGCTATCCTCTGG	ACTCTGACCACTGACTGGAAG
ACTA2	AAAAGACAGCTACGTGGGTGA	GCCATGTTCTATCGGGTACTTC
LOX	GCATACAGGGCAGATGTCAGA	TTGGCATCAAGCAGGTCATAG
LOXL2	AGGACATTCGGATTCGAGCC	CTTCCTCCGTGAGGCAAAC
MMP1	CATGCTTTTCAACCAGGCCC	GTCCAAGAGAATGGCCGAGT
TIMP1	CAACCAGACCACCTTATACC	GAGTGCCACTCTGCAGTTTG
PAI1	CAATCGCAAGGCACCTCTGA	TTCACCAAAGACAAGGGCCA
TGFB1	CTCTCCGACCTGCACAGA	AACCTAGATGGGCGCGATCT

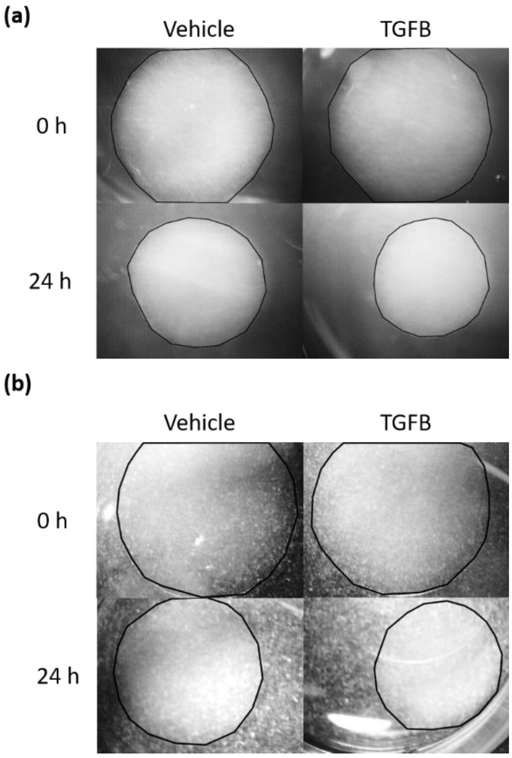

Figure S1: Gel contraction assay in a) iPSC derived cardiac fibroblasts and b) primary adult CF. Related to figure 1.

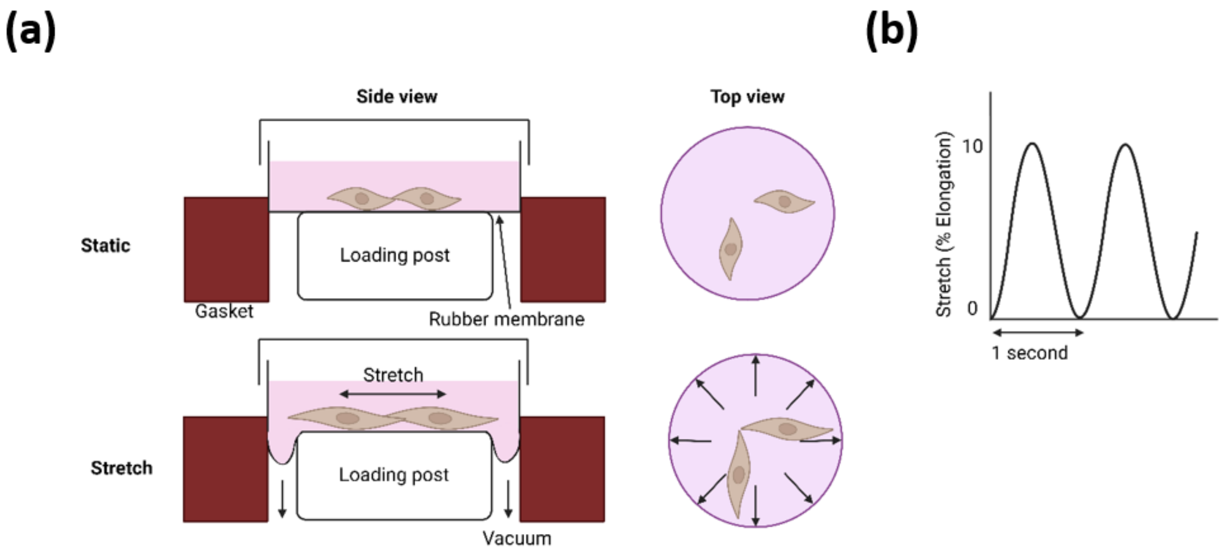

Figure S2: (a) Schematic describing the mechanism of equiaxial cyclic stretch that is applied on the cells using a vacuum system. (b) An example of the sinusoidal stretch pattern applied, with a maximum of 10% elongation and a frequency of 1 Hz. Related to figure 2-5

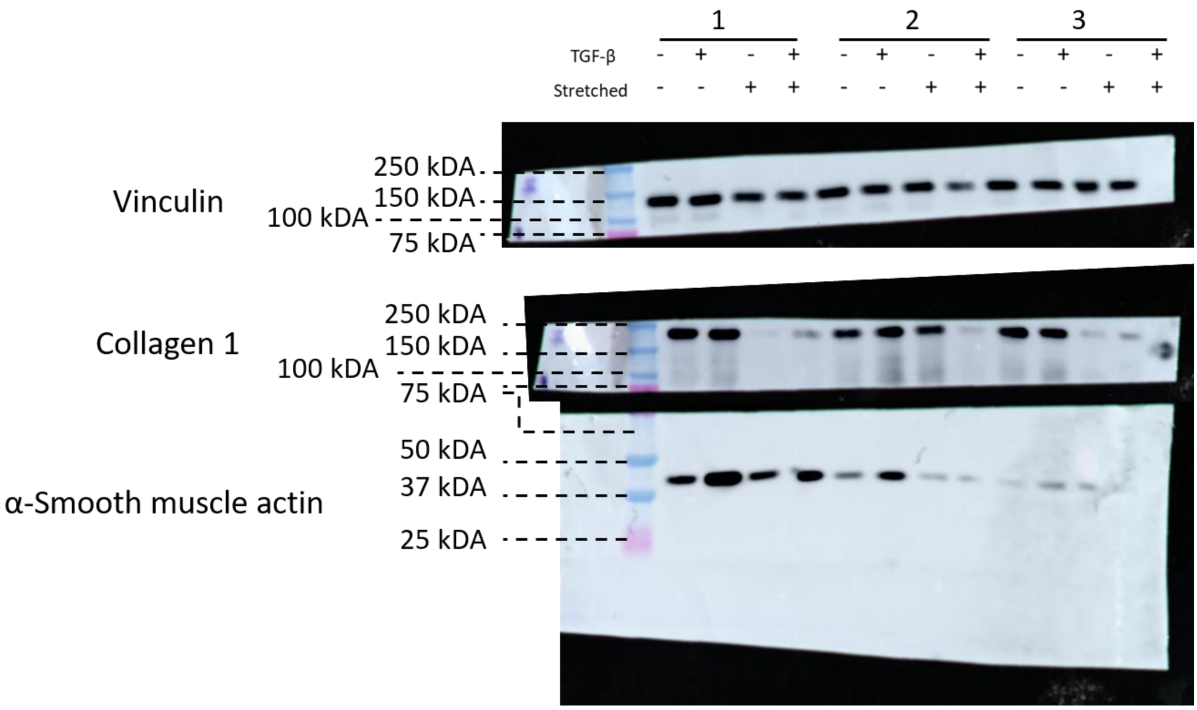

Figure S3: Original blots from western blot analysis for vinculin, collagen 1 and α-smooth muscle actin. Related to figure 2 and 4
